# Giant splenic artery pseudoaneurysm fistulizing to the colon: A case report and focused literature review

**DOI:** 10.1016/j.radcr.2025.12.047

**Published:** 2026-01-27

**Authors:** Tom Simon, Nicolas Brassart

**Affiliations:** a Department of Radiology, Hôpital Universitaire de Bruxelles (HUB) – Université libre de Bruxelles (ULB), Route de Lennik 808, Brussels, 1070, Belgium; b Department of Radiology, Centres Hospitaliers Universitaires HELORA (CHU HELORA), Boulevard John Fitzgerald Kennedy 2, Mons, 7000, Belgium

**Keywords:** Splenic artery aneurysm, Gastrointestinal bleeding, Colonic fistula, Embolization

## Abstract

Splenic artery pseudoaneurysm (SAP) is a rare but dangerous visceral arterial lesion, and fistulization into the gastrointestinal tract is an exceptional and life-threatening complication. We report the case of a 73-year-old woman presenting with acute lower gastrointestinal bleeding caused by a giant SAP that had eroded into the splenic flexure of the colon. Proximal embolization using coils and Onyx achieved initial hemostasis. Although the patient was clinically stabilized, she later developed delayed septic and inflammatory complications requiring splenectomy, colonic resection, partial gastrectomy, and removal of the aneurysmal sac. This case, together with our focused review of published SAP fistula cases, highlights that embolization should be considered a temporizing measure when gastrointestinal fistulization is present; early surgical management remains essential to prevent severe infection and multi-organ complications.

## Introduction

Splenic artery aneurysms (SAAs) are the third most common intra-abdominal aneurysms, but splenic artery pseudoaneurysms (SAPs) differ fundamentally because they lack a complete arterial wall and are far more prone to rupture [1]. SAPs most frequently arise from pancreatitis or inflammatory processes and may erode into adjacent organs. Gastrointestinal fistulization is extremely rare but may result in catastrophic bleeding [2]. Endovascular embolization is widely used as first-line management; however, in cases involving fistulization, its role as definitive therapy remains uncertain [1,3].

We present a case of a giant SAP fistulizing into the splenic flexure of the colon, initially stabilized by proximal embolization but complicated by delayed septic evolution requiring major surgery. A focused literature review is included to contextualize optimal management strategies.

## Case presentation

A 73-year-old woman presented to the emergency room with massive rectal bleeding and abdominal pain. She also reported vomiting without hematemesis and had no prior episodes of rectal bleeding, trauma, vasculitis or history of pancreatitis.

Physical examination showed mild epigastric tenderness. At admission, the patient was hemodynamically stable with a blood pressure of 101/78 mmHg, a heart rate of 80 bpm, and an oxygen saturation of 90% on room air; amylase and lipase levels were normal.

**Fig. 1:**
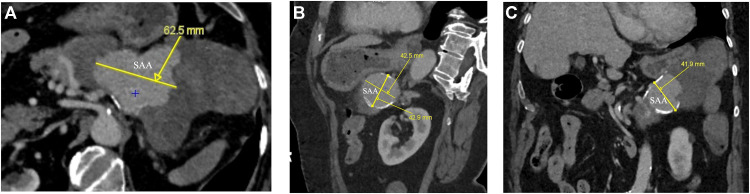
(A) Contrast-enhanced computed tomography (CT), axial multiplanar reconstruction (MPR), demonstrating a large saccular splenic artery aneurysm (SAA), measuring 62 × 42 × 43 mm. (B) Contrast-enhanced CT, sagittal MPR, demonstrating a large saccular splenic artery aneurysm (SAA), measuring 62 × 42 × 43 mm. (C) Contrast-enhanced CT, coronal MPR, demonstrating a large saccular splenic artery aneurysm (SAA), measuring 62 × 42 × 43. CT, computed tomography; DSA, digital subtraction angiography; MPR, multiplanar reconstruction; SAA, splenic artery aneurysm; SAP, splenic artery pseudoaneurysm.

**Fig. 2:**
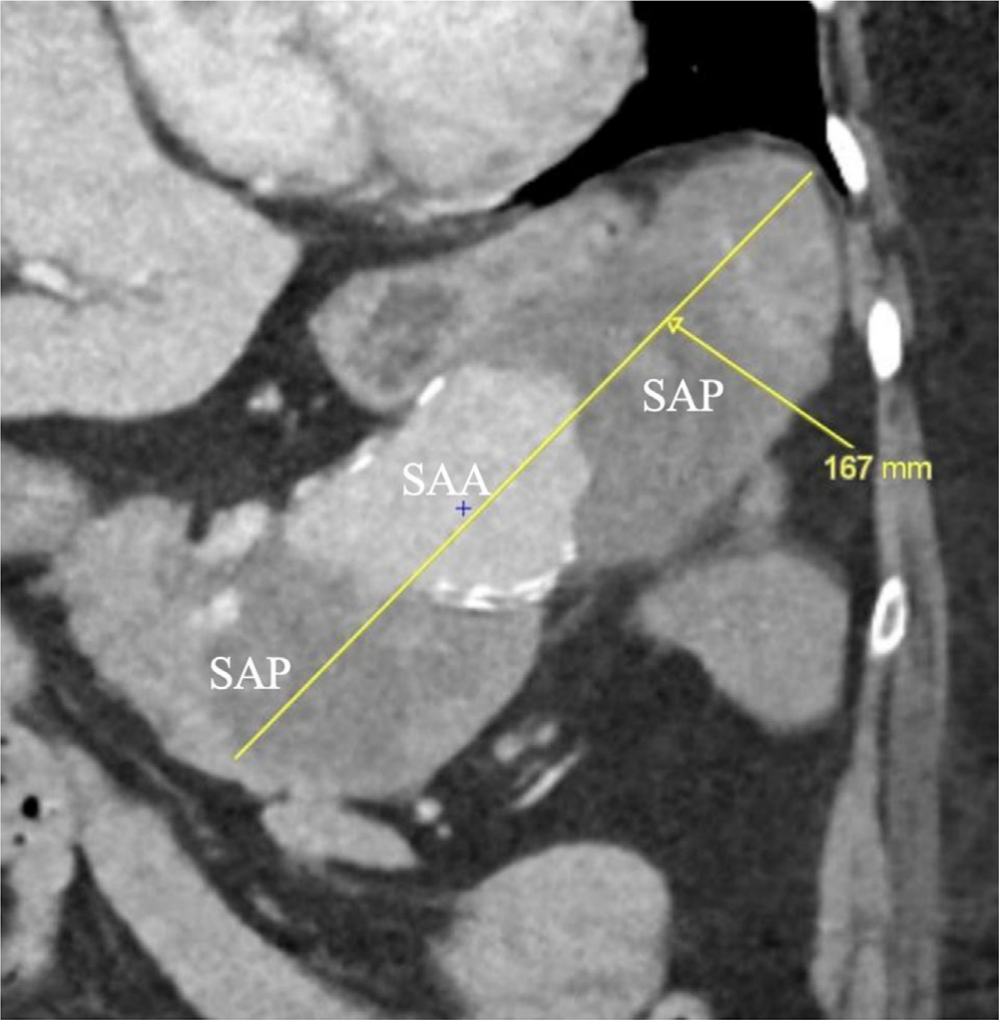
Contrast-enhanced CT, oblique MPR along the long axis of the lesion, demonstrating a giant pseudoaneurysm (SAP) surrounding the saccular splenic artery aneurysm (SAA), measuring ≈16.8 cm in maximal length.

**Fig. 3:**
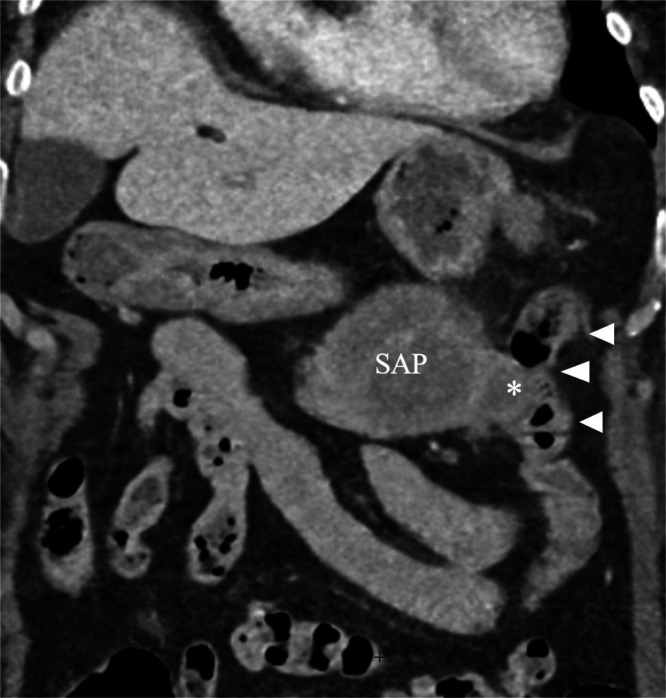
Contrast-enhanced CT, coronal MPR, showing a fistulous tract (asterisk) between the pseudoaneurysm (SAP) and the splenic flexure of the colon (arrowheads).

**Fig. 4:**
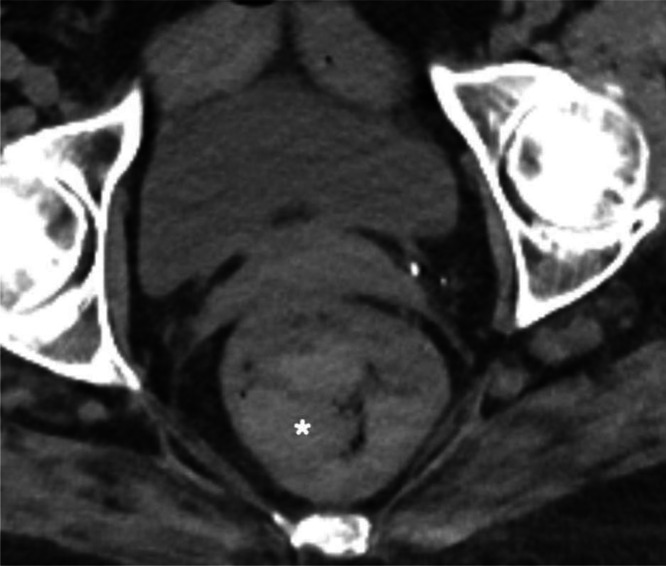
Contrast-enhanced CT, axial pelvis, showing blood clots (asterisk) within the rectal lumen, compatible with active lower gastrointestinal bleeding.

An arterial- and venous-phase contrast-enhanced abdominal CT revealed a large saccular splenic artery aneurysm measuring approximately 42 × 43 × 62 mm (Fig. 1A-C). This lesion was surrounded by an oval-shaped mass containing dense hematic material, partially enhancing on delayed phases, consistent with a pseudoaneurysm measuring approximately 16.8 × 6.5 × 7.3 cm (Fig. 2). The lesion was in contact with the stomach, pancreatic tail, and splenic flexure of the colon, with imaging evidence of active fistulization into the splenic flexure (Figs. 3 and 4).

**Fig. 5:**
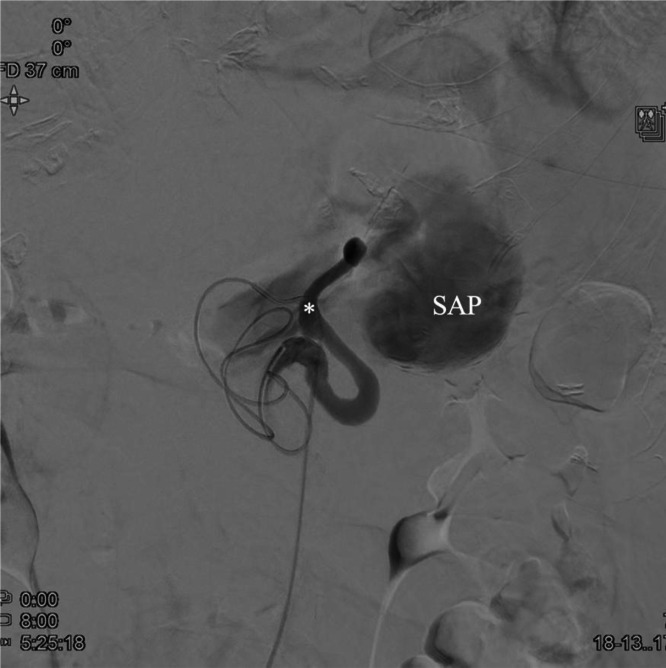
Digital subtraction angiography (DSA), coronal projection, demonstrating the splenic artery (asterisk) and the pseudoaneurysm (SAP) before treatment.

**Fig. 6:**
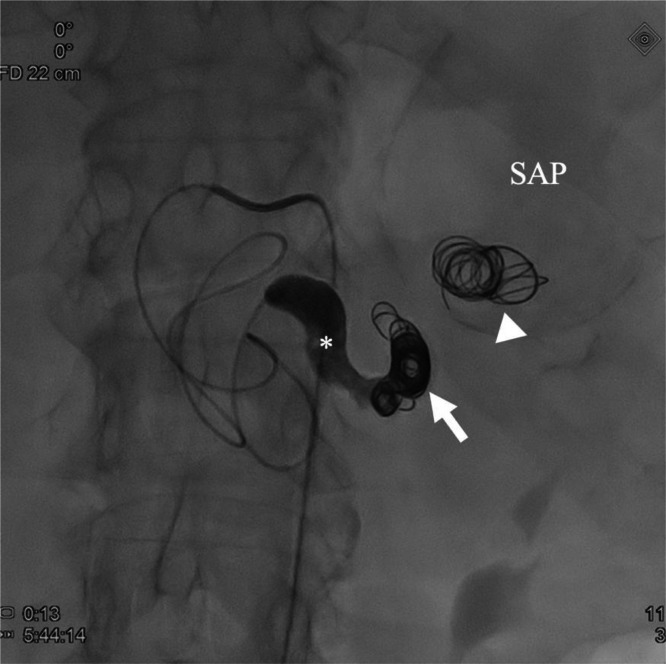
Post-embolization DSA showing proximal embolization of the splenic artery with metallic coils (arrow) and liquid embolic agent Onyx (asterisk) proximal to the pseudoaneurysm (SAP). Migrated coils within the pseudoaneurysmal sac are also visualized (arrowhead).

**Fig. 7:**
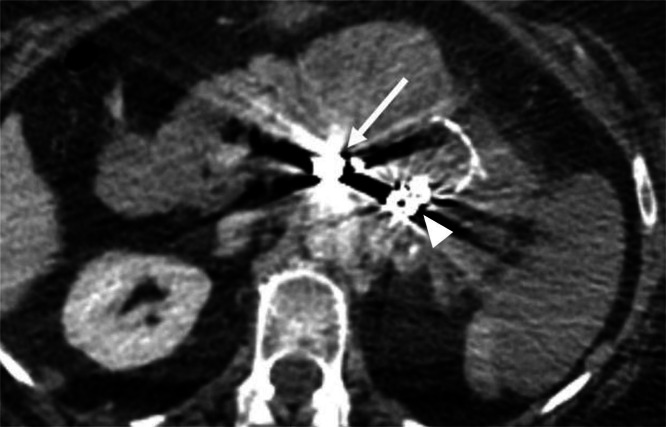
Follow-up contrast-enhanced CT confirming complete exclusion of the pseudoaneurysm (arrow indicates the deployed coils). Coils migrated into the pseudoaneurysmal sac are also visualized (arrowhead).

Urgent digital subtraction angiography confirmed a giant splenic artery pseudoaneurysm with slow wash-out, partial intraluminal hematoma, and sluggish inflow into the distal splenic artery (Fig. 5). Owing to the severely distorted vascular anatomy and the impossibility of identifying a reliable outflow vessel within the aneurysm, distal catheterization and classical “Sandwich” technique embolization were not technically feasible. The initial objective was therefore to exclude the vascular supply of the pseudoaneurysm by proximal embolization. Attempts at dense coil packing within the proximal splenic artery were unsuccessful, as the coils repeatedly migrated into the pseudoaneurysmal sac (Fig. 6), where they failed to provide effective occlusion. Consequently, the embolization strategy was modified to a combined technique using detachable metallic coils (7 × 150 mm) and the liquid embolic agent Onyx. Post-embolization angiography confirmed complete exclusion of the SAP (Figs. 6 and 7).

Due to initial hemodynamic stability and successful embolization, emergency laparotomy was deferred.

A follow-up CT the next day showed near-global infarction of the splenic parenchyma, sparing only the upper pole. Several follow-up CT examinations were performed during the early post-embolization period. A control CT performed on day 5 showed findings similar to those observed on day 1, without progression of the splenic infarction or new collections. On day 16, a new CT demonstrated a splenic subcapsular collection and a small left-sided pleural effusion, while the patient was still clinically asymptomatic, and afebrile, with normal white blood cell counts and stable C-reactive protein levels. Despite the persistence of radiological abnormalities, the favorable clinical course led to hospital discharge.

**Fig. 8:**
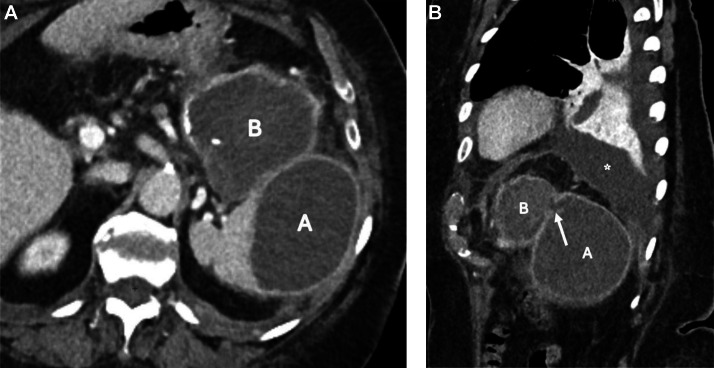
One-month contrast-enhanced CT. Axial image showing splenic subcapsular (A) and perisplenic hematic collection (B) One-month contrast-enhanced CT. Sagittal MPR depicting a fistulous tract (arrow) between the splenic (A) and hematic (B) collections, with associated left pleural effusion (asterisk).

**Fig. 9:**
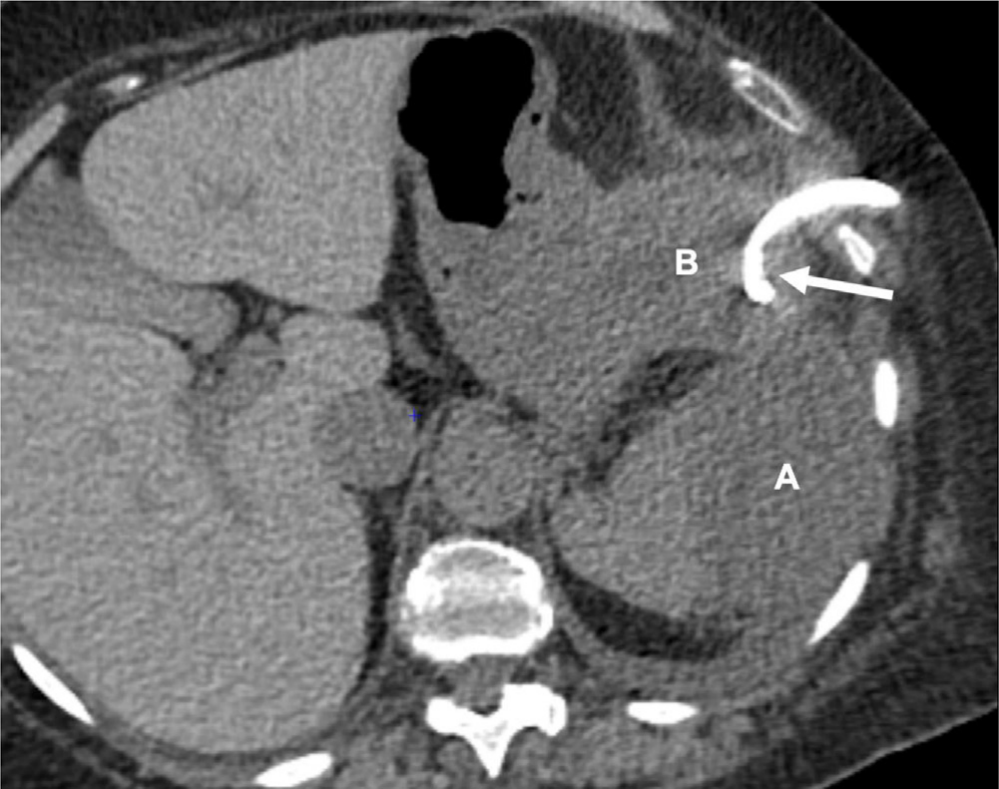
Non-contrast-enhanced CT. Axial MPR, performed after percutaneous drain (arrow) placement in the perisplenic hematic collection (B) fistulized into the splenic collection (A).

One month after embolization, the patient was re-hospitalized with a marked elevation of inflammatory markers, including a C-reactive protein level of 214 mg/L and leukocytosis (11.05 × 10³/µL), despite minimal clinical symptoms. Follow-up imaging allowed better delineation of the fistulous tract between the splenic and hematic collections (Fig. 8), which was clinically suspected given the initial episode of lower gastrointestinal bleeding. Percutaneous drainage of the splenic collection yielded limited hematic and purulent material (Fig. 9), and a separate pleural drain was placed to treat the associated pleural effusion causing dyspnea.

Open surgery was performed, including splenectomy, resection of the splenic flexure, partial gastrectomy, and excision of the pseudoaneurysm sac.

Postoperative complications included septic shock, pancreatic fistula, acute respiratory distress syndrome, and pulmonary embolism.

The patient ultimately recovered and remained well at 1-year follow-up.

**Table:** Timeline of presentation, interventions, and outcomes

Time point	Event	Outcome
Day 0	Emergency admission for massive rectal bleeding and abdominal pain. Contrast-enhanced CT: giant splenic artery aneurysm with surrounding pseudoaneurysm and active fistulization into the splenic flexure → urgent DSA and endovascular embolization (coils + Onyx).	Complete exclusion of the splenic artery pseudoaneurysm; hemodynamic stabilization; emergency surgery deferred.
Day 1	Follow-up CT scan.	Near-global splenic infarction sparing the upper pole.
Day 5	Control CT scan.	Stable findings compared to day 1; no progression of splenic infarction or new collections.
Day 16	Follow-up CT scan.	Splenic subcapsular collection and small left pleural effusion; patient asymptomatic, afebrile, with normal WBC count and stable CRP.
Early post-embolization period	Clinical follow-up during hospitalization.	Favorable clinical course despite persistent radiological abnormalities → hospital discharge.
∼1 month	Re-hospitalization for elevated inflammatory markers (CRP 214 mg/L, leukocytosis). CT: better delineation of fistulous tract between splenic and hematic collections.	Percutaneous drainage of splenic collection; pleural drainage for symptomatic effusion.
Thereafter	Open surgery: splenectomy, splenic flexure resection, partial gastrectomy, and excision of the pseudoaneurysm sac.	Postoperative septic and inflammatory complications.
Postoperative course	Septic shock, pancreatic fistula, ARDS, pulmonary embolism.	Progressive recovery.
1-year follow-up	Clinical assessment.	Patient asymptomatic and in good general condition.

ARDS, acute respiratory distress syndrome; CRP, C-reactive protein; CT, computed tomography; DSA, digital subtraction angiography; SAP, splenic artery pseudoaneurysm; WBC, white blood cells.

## Discussion

Fistulization of splenic artery aneurysms (SAAs) or pseudoaneurysms (SAPs) into the gastrointestinal tract is a rare but life-threatening complication, most often revealed by massive gastrointestinal bleeding [2,[4], [5], [6]]. Pseudoaneurysms are particularly fragile and rupture-prone, and both true and false aneurysms may erode into adjacent structures—especially the colon or stomach—when they reach large dimensions or develop in inflammatory environments such as pancreatitis [1,5,7].

**Fig. 10:**
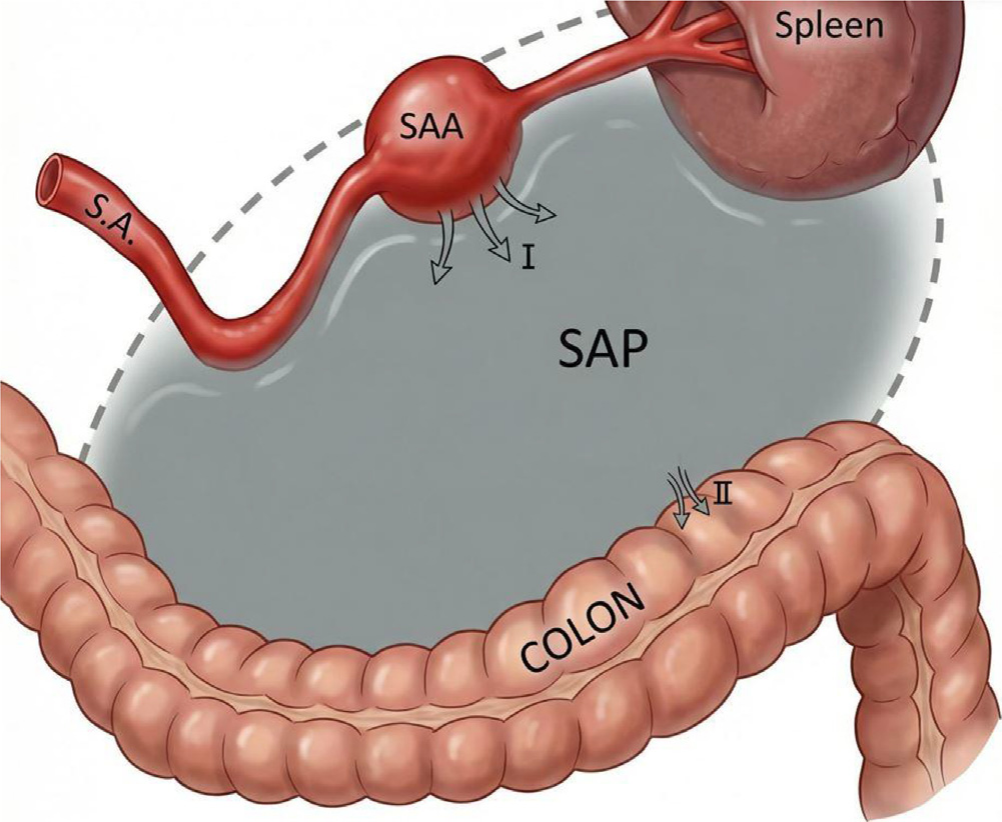
Schematic representation of the proposed pathophysiological mechanism. A true splenic artery aneurysm (SAA) undergoes contained rupture (I), resulting in formation of a large secondary splenic artery pseudoaneurysm (SAP). Persistent inflammatory contact between the SAP and the splenic flexure of the colon leads to progressive erosion and fistulization (II). Proximal embolization may control bleeding but does not eliminate the infected pseudoaneurysmal cavity, explaining delayed septic complications.

From a pathophysiological standpoint, we hypothesize that the lesion initially corresponded to a SAA, estimated at 4.5 cm based on the presence of circumferential mural calcifications. Such calcifications are typically associated with chronic aneurysmal wall remodeling and are less commonly observed in pseudoaneurysms, which lack a native arterial wall. A subsequent contained rupture of this aneurysm likely led to the formation of a splenic artery pseudoaneurysm, which progressively enlarged over an undetermined period. Progressive expansion, local inflammation, and mass effect may have contributed to erosion of the adjacent digestive wall, ultimately resulting in gastrointestinal fistulization (Fig. 10).

In our case, the patient presented with a giant pseudoaneurysm (>10 cm) fistulized into the splenic flexure of the colon, leading to acute lower gastrointestinal bleeding. Emergency endovascular embolization rapidly stabilized the patient and achieved hemostasis, which initially suggested successful management. However, this improvement proved transient: over the following weeks, the patient developed delayed complications, including abscess formation, pancreatic fistula, pleural effusion, and ultimately septic shock.

This unfavorable evolution underscores a crucial lesson: while embolization is highly effective in the acute setting, apparent clinical stabilization should not delay definitive surgical management. In the presence of a digestive fistula, prolonged postponement of surgery allows ongoing inflammation, infection, and local necrosis, which dramatically increase postoperative morbidity.

To better contextualize this timeline-dependent risk, we performed a narrative review of the literature. We searched PubMed and Google Scholar using combinations of the terms “splenic artery aneurysm,” “pseudoaneurysm,” “fistula,” “colon,” “stomach,” “small bowel,” “digestive tract,” and “gastrointestinal bleeding,” identifying 19 relevant cases of splenic artery (pseudo)aneurysms fistulizing into the digestive tract. Our review (Table 1) highlights distinct patterns based on the fistula location: the stomach remains the most common site (*n* = 12), followed by the colon (*n* = 6) and the small bowel (*n* = 1).

**Table 1:** Review of relevant published cases of splenic artery (pseudo)aneurysms with digestive fistulization.

Author (Year)	Type	Fistula location	Aneurysm size	Initial management	Definitive management	Outcome
Colonic fistulas						
Sentenach et al. (1997) [8]	SAA	Splenic Flexure	4 cm	None (Emergency)	Open surgery (Splenectomy + Colectomy)	Uneventful recovery.
Sakai et al. (2016) [9]	SAP	Transverse Colon	10 cm	Transfusion	Open surgery (Splenectomy + Colectomy)	Recovered. Complex course (pancreatitis).
Maharaj et al. (2018) [10]	SAA	Splenic Flexure	6 cm	None (Emergency)	Open surgery (En-bloc resection)	Uneventful recovery. Symptom-free at 2 years.
Costa et al. (2021) [4]	SAP	Transverse Colon	NR	Endovascular (Coils)	None (Conservative follow-up)	Uneventful recovery. No rebleeding.
Zogheib et al. (2024) [5]	SAA	Stomach + Colon	10 × 12 cm	None	Open surgery (Splenectomy + Repair)	Uneventful recovery.
Present case	SAP	Splenic Flexure	16.8 cm	Endovascular (Coils + Onyx)	Open surgery (Splenectomy + Resections)	Survival after septic shock and ARDS.
Gastric fistulas						
AbuRahma et al. (1991) [6]	SAA	Stomach	NR	None	Open surgery (Splenectomy + Resection)	Uneventful recovery.
Chiu et al. (1993) [11]	SAA	Stomach	Giant (> 5 cm)	None	Open surgery (Splenectomy + Gastrectomy)	Uneventful recovery.
Ghassemi et al. (2010) [12]	SAA	Stomach	4 cm	None	Open surgery (Gastric suture)	Uneventful recovery.
Al-Habbal et al. (2012) [13]	SAA	Stomach	8 cm	None	Open surgery (Splenectomy + Gastrectomy)	Uneventful recovery.
Ma et al. (2013) [14]	SAA	Stomach	5 cm	None	Open surgery (Splenectomy + Repair)	Uneventful recovery.
Yang et al. (2013) [15]	SAA	Stomach	4.8 cm	None	Open surgery	Uneventful recovery.
Akbulut et al. (2015) [1]	SAA	Stomach	Giant (> 5 cm)	Emergency laparotomy	Splenectomy + Partial gastrectomy	Death. Intraoperative cardiac arrest.
Yue et al. (2015) [16]	SAP	Stomach	0.6 cm	None	Open surgery (Ligature)	Recovered after ARDS.
Karabulut et al. (2016) [17]	SAA	Stomach	Giant (> 5 cm)	Resuscitation	Surgery (Attempted)	Death. Massive hemorrhagic shock.
Fierro et al. (2017) [18]	SAP	Stomach	NR	None	Open surgery	Uneventful recovery.
Panzera et al. (2020) [2]	SAA	Stomach	>5 cm	Endoscopy (Glue)	Open surgery (Splenectomy + Pancreatectomy)	Good outcome.
Ben Layne et al. (2021)	SAP	Stomach	NR	Endoscopy (Clip)	Angioembolization + Surgery	Recovered. Prolonged hospital stay.
Small bowel fistulas						
Chen et al. (2021) [19]	SAP	Jejunum	NR	None	Open surgery (Resection)	Recovered. Diagnosis was difficult.

ARDS, acute respiratory distress syndrome; NR, not reported; SAA, splenic artery aneurysm; SAP, splenic artery pseudoaneurysm.

Analysis of these cases reveals that while gastric fistulas are more frequently reported, they are associated with a high risk of massive hemorrhage and mortality if not surgically managed immediately. Conversely, colonic fistulas—such as the one presented here—are exceptional.

The literature supports our observation: embolization alone may suffice for small, contained aneurysms (<5 cm) without overt fistula, but in giant aneurysms (>5 cm) with digestive tract erosion, a 2-step approach is consistently favored. Immediate endovascular control is valuable for hemodynamic stabilization but must be rapidly followed by early surgical resection to prevent delayed complications [[3], [4], [5], [6]].

Recent expert consensus and systematic reviews have attempted to standardize the management of visceral artery aneurysms (VAAs), focusing on size thresholds and symptomatology. The 2023 ESVS clinical practice guidelines recommend intervention for SAAs >2 cm or in the presence of symptoms or pseudoaneurysmal morphology, but do not address fistulization into hollow organs [20]. Our case highlights this gap and underlines the need for management strategies that explicitly incorporate gastrointestinal fistulization as an indication for expedited surgery.

## Conclusion

For splenic artery pseudoaneurysms complicated by gastrointestinal fistula, embolization is an essential first step for hemorrhage control but should be considered a temporizing measure only. Definitive surgical management should follow within days to remove the infected and necrotic focus and prevent delayed septic and inflammatory complications. Early multidisciplinary planning is crucial in these complex cases.

## Data availability

All data are included in this article; additional de-identified information is available upon reasonable request.

## Patient consent

Written informed consent was obtained from the patient for publication of this case report and accompanying images. Identifiers were removed and images anonymized in accordance with journal policy.
